# Utilising LiDAR for fall detection

**DOI:** 10.1049/htl2.12001

**Published:** 2021-01-20

**Authors:** Nikolai Frøvik, Bashir A. Malekzai, Knut Øvsthus

**Affiliations:** ^1^ Department of Computer Science Electrical Engineering and Mathematical Sciences Western Norway University of Applied Sciences Bergen Norway

## Abstract

Autonomous driving generates several low‐cost technologies, such as light detection and ranging (LiDAR). Due to this, the LiDAR technology has experienced impressive performance improvements. Our ambition is to capitalise on this development, where LiDAR is considered as the enabling technology for a non‐invasive monitoring system for securing elder persons in their home. A motivation for technology‐based securing of elder persons is that many countries experience a demographic change. Traditional personal care by care worker or re‐location to special homes of elder persons does not scale due to the shrinking fraction of the working population. Technology can reduce some of the burden. This article proposes and assesses technology for securing a person's home. However, securing a person, based on monitoring, requires careful design because the technology should be non‐invasive, reliable and low cost. LiDAR technology offers several crucial qualities that meet these system requirements. This article provides a proof of concept for a low‐cost, non‐invasive LiDAR‐based monitoring system. Our proposed system can detect if a person has fallen, and it can trigger an alarm to the care services when required. We emphasise especially that our monitoring solution can operate in the bathroom and even in the shower.

## 1 INTRODUCTION

The population of many developed countries are ageing, resulting in a decreasing workforce relative to the part of the population being retired [1]. In order to meet the altering demand and maintain service quality, the care services must be redesigned. People subjected to physical and cognitive decline can be safeguarded by technology and thus enable them to live longer at home. This can be done by monitoring the user by means of various sensors in order to detect dangerous situations in progress, lifestyle changes and emergencies that require immediate response. In order for such monitoring systems to be accepted, two significant factors are the intrusiveness of the technology and the privacy concerning the details in the acquired information [2]. Consequently, the monitoring systems should preferably be small, discreet and not acquire more details about a user than necessary for the intention of the system. For example, in order to tell if a user has fallen or if a change in gait speed has occurred over time, a video camera provides a lot more information than needed; hence, it classifies as intrusive. Fall, for example, frequently occurs in the bathroom, where cameras are considered too intrusive.

Formerly, light detection and ranging (LiDAR) technology has been known to be expensive and power demanding [3]. However, recently, significant progress has made LiDAR sensors cheaper, smaller and more power efficient [4, 5, 6]. As a result, LiDAR technology is becoming a promising choice when developing monitoring systems [7].

This article proposes and demonstrates a LiDAR sensor monitoring prototype developed by the authors. The system design is based on the design factors in [2], and the goal is to investigate the applicability of LiDAR used as a monitoring system. The article first presents related work in Section 2, followed by a short technical presentation of the system in Section 3. Section 4 presents the tests and results. Section 5 discusses the results. Finally, Section 6 concludes this paper.

## 2 RELATED WORK

To the best of our knowledge, we have not seen any similar LiDAR‐based monitor systems. Several monitoring systems have been designed for the purpose of making users feel safer and providing a higher degree of independence in their own home environment. Existing monitoring systems have been realised utilising a variety of sensing technologies. Existing solutions include wearable and non‐wearable systems [8].

Wearable systems are often based on a pendant or wristband with sensors such as accelerometers, gyroscopes and barometric pressure sensors [9, 10]. The former two detect acceleration and angular velocity, respectively, allowing falls to be detected if a sudden change in motion occurs, e.g. if a person trips and falls to the ground. For the latter sensor, it detects fall by measuring the change in the barometric pressure between waist height and floor level. The two main challenges, related to wearable sensors, are that the older people often follow a slow trajectory as they fall (no hard impact), and the person has to remember to wear the unit.

Non‐wearable systems are sensor technology installed in the user's home environment (or other place of interest). If such systems are designed properly, the user can live her/his normal life, and it will have a limited visual impact in the home. Sensor technologies used in such systems are, among other, infrared depth cameras [11], radar [12, 13], ultrasound [14], intelligent carpet (light transmission of light in optical fibres, built into carpet) [15], microphones [16] and cameras [17]. Our proposed LiDAR‐based solution fits well into these technologies and offers attractive complementary features such as small size, non‐invasive, and well suited for use in places where intimate situations occur.

Automated driving systems [4, 18] and robotics [19] demand high‐precision vision systems. These two areas are the main research areas challenging the industry to increase the LiDAR's capability and reduce the price. Several sensors are required to provide autonomous driving and operations [20]. LiDAR is a promising candidate. It produces excellent three‐dimensional point clouds, a necessity for measuring distances to objects and proximity detection. Furthermore, the mass production of units will cause a low unit price.

## 3 THE MONITORING SYSTEM

Due to the recent advancement of LiDAR technology, we consider LiDAR a promising sensor for making a monitoring system, e.g. for homes of elderly people. Such a monitoring system is able to monitor daily activity patterns as well as emergencies where immediate response is needed. While holding the functionality as described, such a monitoring system can also be made small and non‐invasive, i.e. not introducing significant aesthetic or practical changes where this system is installed. Next, we describe how the proposed monitoring system could be realised.

**FIGURE 1:**
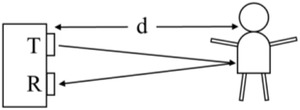
LiDAR sensor transmitting (*T*) a light pulse towards an object. Some of the reflected light is received (*R*) by the sensor, enabling a measurement of distance (*d*) between the sensor and the object

The LiDAR sensor used in this paper (VL53L1X [21]) uses time of flight of a light pulse to estimate the distance between the sensor and an object, e.g. a human, Figure 1.

**FIGURE 2:**
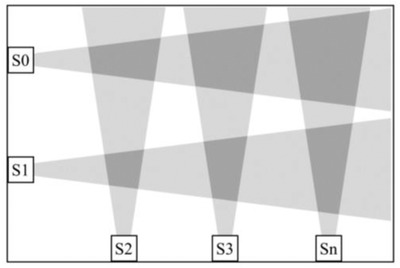
Monitoring concept illustrated by using five LiDAR sensors to coverer a room. The light grey areas illustrate the FoV of the sensors. The dark grey area is the area in which two sensors overlap

The LiDAR has a relatively narrow field of view (FoV), approximately 20–30°; hence, one sensor is not able to cover an entire room. However, by combining multiple LiDAR units in one room, the entire area of a room can be covered, as illustrated in Figure 2. With a network of sensors, like that seen in Figure 2, an analysis of the acquired data can provide important parameters regarding a person. Parameters of interest include gait speed and falls. Gait speed is important as it can be used as a predictor of cognitive decline, falls, institutionalisation and survival in elderly people [22, 23].

Falls can be detected by analysing presence and lack of presence inside a room. For example, if the sensors monitoring the room is placed at chest height, and additional sensors are placed in every entrance to the room, one can conclude that a fall has occurred if a person is no longer observed inside the room (chest height), and there has not been detected movement in any of the room's exits/entrances. Notice that alarms are forwarded to the care service; thus, the personnel will investigate the situation. The healthcare service would not just be notified in the case of a sudden fall, but also if the resident was not able to stand up after intentionally crouching to reach for something on the floor. Hence, regardless of the incident resulting in the person laying on the floor, the healthcare services can be notified so that a long lie can be prevented. Long lies have serious negative impact on a person's health and may cause an increased chance of dying within a year following the accident [24]; it is, therefore, essential to prevent such incidents.

**FIGURE 3:**
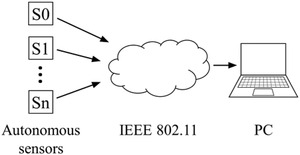
Schematic drawing of measurement system. Multiple autonomous sensors sends distance measurements to a computer through a LAN

To demonstrate and validate the monitoring system, a prototype has been made, consisting of two sensor units, and a PC collecting data from the two sensors through a hotspot Wi‐Fi. The system is schematically illustrated in Figure 3.

**FIGURE 4:**
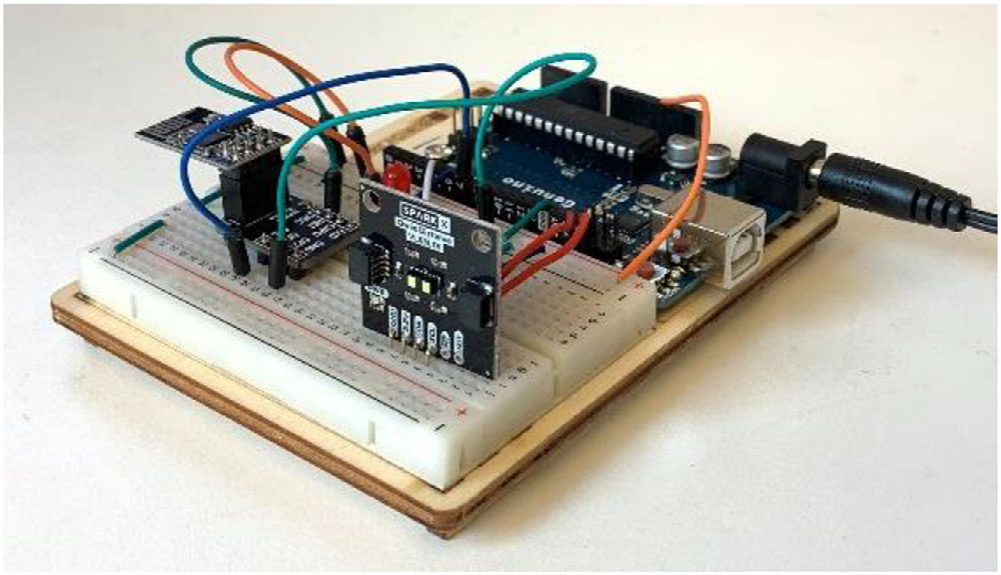
Sensor unit consisting of a microcontroller, LiDAR sensor and Wi‐Fi module

The sensor units (S0, S1 etc.) consist of an Arduino Genuino (UNO) microcontroller, a VL53L1X LiDAR‐sensor supplied by SparkFun and an ESP8266 Wi‐Fi module allowing the microcontroller to connect to an IEEE 802.11 network for wireless data transmission. The sensor unit can be seen in Figure 4. IEEE 802.11 was chosen because appropriate hardware with associated application programming interface (API) was easily accessible and it is a low‐cost alternative. Communication between the sensor and the microcontroller uses an inter‐integrated circuit. SparkFun provides an API making it easy to configure and read data from the sensor through the Arduino environment. In addition to distance readings, the LiDAR reports the validity of its measurement. A valid measurement is indicated by the number zero; otherwise, the measurement status will be an integer, larger than zero, indicating a problem with the measurement. The microcontroller is programmed to continuously transmit UDP packages to the PC, containing a distance reading and the validity or status of the current distance reading. When a data packet is received by the computer, its data are stored in an array in addition to a timestamp (time received).

The sensor reading is not synchronised between the sensors. Thus, the data presented differ slightly in observation time. An important design goal is the simplicity of the system; avoiding time‐synchronised units reduces the cost and helps scaling the system to include more sensors, as shown in Figure 2. We found that synchronisation was not critical as the person's movement is slow compared to the time difference between measurements from the two sensors. This topic will be further discussed and analysed in our experiments in the following section.

## 4 SYSTEM PERFORMANCE

Several experiments have been carried out in order to characterise some key parameters of the detection system. This section presents the experiments and the results.

### 4.1 Measurement range and accuracy

Measurement range and accuracy were investigated by performing several distance measurements at known distances. The two sensors were placed 200 mm apart, oriented in the same direction, on top of a trolley, 920 mm above the floor. The trolley was carefully placed at 18 different distances between 20 and 6000 mm away from the wall. The reference distance was measured with a metre (1‐mm resolution). For every position, the system acquired about 100 distance measurements from each sensor. Mean distance for every length interval was then calculated. To reduce the error introduced by manually positioning the sensors at different distances from the wall, measurements for the 18 distance intervals was repeated five times. From the five repetitions, the mean distance was calculated. The standard deviation provides an estimate of the measurement uncertainty.

**FIGURE 5:**
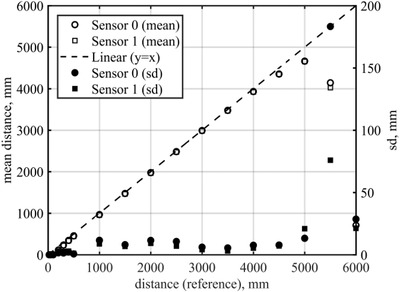
Distance measurements (non‐filled) between 20 and 6000 mm. Each data point is the mean of five experiments. Filled circles and squares are one standard deviation of the mean distance (right *y*‐axis)

The mean distance is plotted as a function of the reference distance in Figure 5. All data presented in the range 20–3000 mm had a valid status code. At 3500 mm, the fraction of valid measurements was 34% and 42% for sensor S0 and sensor S1, respectively. At 4000–5500 mm, there were no valid measurements for any of the sensors. Finally, at 6000 mm, sensors S0 and S1 had 2% and 4% valid measurements, respectively. The measurements with non‐valid status have not been investigated further for this experiment. Section 4.2 presents a more detailed assessment of the data reported as invalid.

### 4.2 Field of view

**FIGURE 6:**
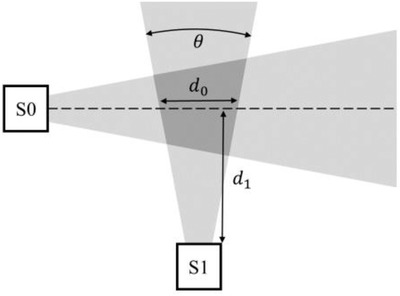
Experimental setup of sensors for determining the sensors FoV (θ). A person walked the dashed line from left to right. $d_{0}$ is the distance the person was within the FoV of sensor S1 with the closest shoulder being a distance, $d_{1}$, from the sensor. The light grey areas illustrate the FoV of the two sensors. The dark grey area is the area in which the two sensors overlap

The LiDAR sensors ability to cover an area depends on two parameters, the range in which the sensor obtains reliable distance measurements and the sensors FoV. To investigate the sensors’ FoV, several experiments were conducted using the setup seen in Figure 6.

A person walked along the stapled line from left to right (see Figure 6), with a mean velocity of v = 0.7 ± 0.2 m/s for these experiments, which is a realistic gait speed for elderly people [23]. This was repeated 10 times for each of the three distances of 1, 2 and 3 m between the subject's shoulder and sensor S1 ($d_{1}$), resulting in a total of 30 sets of measurements. The sampling rate of the two sensors is not synchronised, so that measurements from the two sensors are obtained with a few milliseconds separation. However, we observed that the sampling rates of the two sensors are comparable and stable for both sensors (as seen in Table 2). Hence, all the measurements from sensor S0 could be paired with a measurement from sensor S1 with only a minor time difference. Such a pair of distance measurements, one from sensor S0 and one from sensor S1, with a minor time difference (typically less than 43 ms), will from now on be referred to as a data point. *d*_S0_ will be the *x*‐coordinate and *d*_S1_ is the *y*‐coordinate.

**FIGURE 7:**
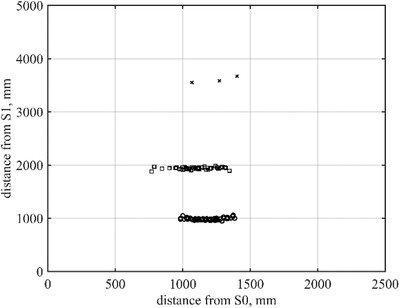
Distance from sensor S1 plotted as a function of the distance from sensor S0. Figure 4 shows the experimental setup used. A person walked a straight line in front of sensor S0, at a distance approximately 1 m (circle), 2 m (square) and 3 m (cross) away from the sensor. Only data points where both sensors reported valid data are plotted

The valid data points from the 30 experiments (walking the dashed line in Figure 6 one time) were plotted in Figure 7, with circle markers for the sets at 1 m from sensor S1, square for 2 m and cross for 3 m. When the person is positioned on the far left or right of the sensor, the data from the LiDAR are marked as invalid and can be filtered out as in Figure 7. Eventually, when the person transition into the view of the sensor, the measurements become valid.

Based on valid data points only, the sensor's FoV was calculated to be 17.8 ± 0.1° for the experiments conducted at 1 m from sensor S1 and 19.3 ± 0.1° at 2 m. At 3 m, most measurements were invalid, and only three data points were obtained.

### 4.3 Bathroom environment

The bathroom is the place where our most intimate activities of daily living occur. Hence, a monitoring system that produces detailed images is even more invasive in the bathroom compared to other areas in the home. The bathroom is, therefore, an interesting location for a LiDAR‐based monitoring system, as the data produced are less invasive than a camera or an infrared depth camera.

**FIGURE 8:**
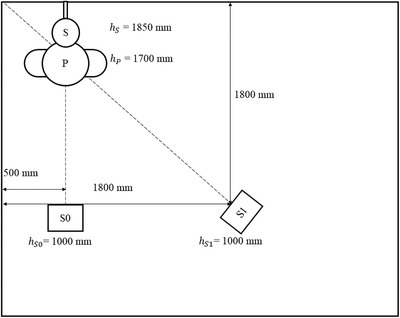
Bathroom environment. S0 and S1 are the two sensors, P is the person present during some experiments, and S is a showerhead attached to the wall

In order to cover all activities in a bathroom, a monitoring system must perform well, even when the user is taking a shower. Because of this, the system performance was investigated by acquiring measurements in a typical shower setting. The experimental setup can be seen in Figure 8. The sensors were placed in such a way that the light beam crossed the shower's water jet and reflected from the person. This enabled the detection of the person standing underneath the showerhead.

The experiments aimed to investigate the sensor's ability to discriminate between a human and a water jet. Without this ability, dangerous events, such as falls, can occur unnoticed if a person taking a shower is occluded by the water jet.

First, a reference experiment was conducted without anyone in the shower, and without any water jet (experiment 1). Afterwards, measurements was performed while the water was running and without any person standing in the shower (experiment 2). No significant changes were observed between these two experiments, as can be seen from Table 1.

**TABLE 1:** Results from experiments conducted in a shower environment

Exp. no.	*d*_S0_ [mm]	*n*_S0_ (valid)	*n*_S0_	*d*_S1_ [mm]	*n*_S1_ (valid)	*n*_S1_
1	1758 ± 3	25	26	2404 ± 6	25	25
2	1755 ± 3	31	32	2398 ± 5	31	33
3	1408 ± 19	55	56	2059 ± 12	53	56
4	1490 ± 7	43	45	2121 ± 8	44	44
5	1429 ± 9	51	53	2123 ± 15	49	54
6	1427 ± 7	44	45	2102 ± 12	40	45

Next, four experiments with different configurations was performed while a person (*h* = 1700 mm) stood in the shower. First, the person stood underneath the showerhead with no water running (experiment 3). Second, the water was turned on while the person remained in place (experiment 4). Thereafter, the person grabed the showerhead by his hand and held it approximately 200 mm (experiment 5) and 400 mm (experiment 6) in front of his body, in the direction of sensor S1. The showerhead was held a bit higher than the height of the person, so that most of the body was behind the water jet as seen from sensor S1's perspective.

Regarding experiments 4–6, the sensors produced concistent distance readings, even though a water jet was placed to occlude the person in experiments 5 and 6. For sensor S0, there was a slight difference of about 60 mm from experiment 4 to experiments 5 and 6. This difference is likely due to the slight rotation of the person towards sensor S1 when holding the showerhead in this direction.

In Table 1, *d* is the distance represented by mean ± standard deviation. The subscript indicates the sensor ID. *n* is the number of measurements for the respective experiment and sensor, and *n* (valid) is the number of valid measurements. Calculation of mean and standard deviation is based on valid measurements only.

### 4.4 Sampling performance

As for any monitoring system, the time response must be adequate for monitoring elderly people. Adequate temporal resolution imposes requirements on the sampling frequency, in order to ensure that relevant information is not missed. The sampling performance of the measurement system was investigated by analysing the measurements from all experiments described in Sections 4.1–4.3. Table 2 contains mean time between two consecutive measurements for both sensors in each experiment, both for all measurements, and for valid measurements only.

**TABLE 2:** Sampling rate during each experiment category

	Valid measurements		All measurements	
Experiment category	*Δt* [ms]	*n*	*Δt* [ms]	*n*
Accuracy (4.1), S0	94 ± 113	7183	87 ± 7	10,532
Accuracy (4.1), S1	95 ± 129	7237	87 ± 7	10,528
FoV (4.2), S0	90 ± 27	1506	87 ± 7	1843
FoV (4.2), S1	114 ± 75	85	87 ± 5	1828
Shower (4.3), S0	175 ± 23	542	174 ± 16	558
Shower (4.3), S1	181 ± 39	527	174 ± 17	558

As seen in Table 2, the sampling rate (1/*Δt*) for valid measurements only differed in the measurement environment. The experiment setup in Section 4.2 for sensor S1 resulted in many invalid measurements, hence resulting in a large *Δt* (both mean and standard deviation) for valid measurements only compared to the *Δt* based on all measurements, including both valid and invalid.

Regarding the experiments conducted in the shower, the shower wall was within the sensor range, resulting in mostly valid measurements (97% valid for sensor 0 and 94% valid for sensor 1). Consequently, only a small difference was seen between *Δt* for all measurements and valid measurements only.

As stated above, *Δt* is the time difference between two consecutive measurements, as they are timestamped at the PC. The time difference includes the configured periodic measurement interval, the transmission time, and processing time at the PC. The column where all measurements are presented shows consistency between the two sensors in the same environment. Regarding *Δt* in the shower experiments being twice as much as for the other tests, this is likely due to the change of location for the experiments. With different interfering traffic, this is likely to affect the monitoring system's data transmission performance.

In Table 2, *Δt* is the time between two consecutive measurements for one sensor. For the three experiment categories in the table, mean, standard deviation and *n* have been calculated based on the measurements from all experiments within the respective categories. The number in the parenthesis refers to which subsection the main results of the respective experiment are found.

Additional experiment was conducted to investigate the significance of transmission time between the sensors and the computer. An experiment was designed where a piece of cardboard (0.8 × 0.6 m^2^) was held at about 1.5 m in front of the two sensors, with a wall approximately 1.5 m behind the cardboard (about 3 m from the sensor). While measuring, the cardboard was pulled rapidly away from the sensors’ FoV, doubling the measured distance. At the moment, when the cardboard was pulled away, a button was pushed on the computer, tagging the next incoming measurement. This way, the transmission time was calculated as the time difference between the tagged measurement and the moment the distance readings changed from about 1.5 to about 3.0 m. Clearly, the experiment involves several uncertainties, such as the movement of the board and the push on the bottom. Therefore, several repeated experiments were conducted to reduce the significance of these uncertainties.

The experiment described above was performed 10 times, resulting in a mean time difference of 135 ± 89 ms for sensor S0, and 130 ± 90 ms for sensor S1. Considering the manual way of conducting the experiment (pressing the button and removing the cardboard) and a mean time between the received measurements of 88 ms, one should probably add about a quarter of a second of uncertainty to the experiment. Even when taking the last considerations into account, the time from the moment the cardboard is pulled out of the sensor's FoV until the measured distance is changed does not exceed 0.5 s.

## 5 DISCUSSION

In the preceding section, some key characteristics of the proposed monitoring system have been investigated through various experiments. We now return to the system design, as shown in Figure 2, and discuss our findings from the perspective of designing a system for securing a person's home. Figure 2 illustrates one of several potential sensor arrangements. Clearly, the arrangement depends on the requirements.

The shower/bathroom scenario documents a reliable, non‐intrusive solution. Currently, technical solutions designed for independent showering for fragile older persons are appearing [25, 26]. However, a remaining challenge is to ensure the safety of the person in the shower and detect if the person has fallen. We find that our LiDAR‐based solution detects the presence of the person in the shower, and it can trigger an alarm when required. Furthermore, we find that the capability of identifying the person and responding to absence is adequate to secure the person. The shower scenario demonstrated the robustness of the LiDAR‐based solution concerning water and moisture. Furthermore, LiDAR‐based systems are currently investigated in the harsher outdoor environment [7] that is far more challenging than homely environments. The final solution will be contained in a box with a window that may require periodic cleaning. In the case that dust degrades the signal, the degradation can trigger a warning that cleaning is required.

Covering larger rooms in the home requires more sensor than in the bathroom. An important design goal for the system was low cost per unit; this enables the use of several autonomous sensors. However, synchronising the sensor may impose limitations on the scalability of the system in terms of the number of sensors. Our investigations demonstrate that the system provides a satisfactory time response. The main reason is that the movement of a person is slow (typically in the range 0.4–1.6 m/s [23]) compared to the achieved response of the system.

The measurement range of the LiDAR sensor used is sufficient for many locations, especially smaller rooms, which is often the case for bathrooms. The number of suitable locations for using this system can be extended further by using a sensor with increased range. However, one must keep in mind that extended sensor range will generally involve the trade‐off of increased power consumption, unless the increased range is the result of technological improvements rather than increased power of the transmitted light.

The communication protocol used for this prototype was adequate for early testing of the concept/system. However, for an end product, a communication protocol that is more appropriate for sensor networks should be chosen. IEEE 802.11 is not optimal regarding power efficiency and transmission rate stability, as competing Wi‐Fi communication may alter the sampling performance. Communication protocols such as IEEE 802.15.4 or Bluetooth Low Energy should be considered for future improvements. The final success factor of the system depends on its unit cost. Based on experience working with alternative radar solutions [12, 13] and price for radar systems found in [27], we estimate that the solution will have a unit price less than $10. The estimate is based on publicly available unit prices on the Internet.

## 6 CONCLUSION

A low‐cost LiDAR system is demonstrated for monitoring and securing an elder person's home. The bathroom is the area in a home with high risk of falling. The bathroom is a well‐suited environment for using our sensor solution because of its ability to detect a person taking a shower—the water will not occlude the person. Furthermore, our system offers a monitoring solution that is non‐invasive, capable of detecting whether a person has fallen in the shower. Several independent LiDAR units are required to cover an entire room. Our investigations find that each unit can be designed with low‐cost components that transmit their data to a local hub for evaluation. Synchronisation of the units is, based on our findings, not required.
